# Acute and Chronic Sustained Hypoxia Do Not Substantially Regulate Amyloid-β Peptide Generation *In Vivo*

**DOI:** 10.1371/journal.pone.0170345

**Published:** 2017-01-18

**Authors:** Alberto Serrano-Pozo, Manuel A. Sánchez-García, Antonio Heras-Garvín, Rosana March-Díaz, Victoria Navarro, Marisa Vizuete, José López-Barneo, Javier Vitorica, Alberto Pascual

**Affiliations:** 1 Department of Neurology, University of Iowa Hospitals & Clinics, Iowa city, Iowa, United States of America; 2 Instituto de Biomedicina de Sevilla (IBiS), Hospital Universitario Virgen del Rocío/CSIC/Universidad de Sevilla, Seville, Spain; 3 Departamento de Bioquimica y Biologia Molecular, Facultad de Farmacia, Universidad de Sevilla, Seville, Spain; 4 Centro de Investigación Biomédica en Red sobre Enfermedades Neurodegenerativas (CIBERNED), Spain; Torrey Pines Institute for Molecular Studies, UNITED STATES

## Abstract

**Background:**

Recent epidemiological evidence has linked hypoxia with the development of Alzheimer disease (AD). A number of *in vitro* and *in vivo* studies have reported that hypoxia can induce amyloid-β peptide accumulation through various molecular mechanisms including the up-regulation of the amyloid-β precursor protein, the β-secretase Bace1, or the γγ-secretase complex components, as well as the down-regulation of Aβ-degrading enzymes.

**Objectives:**

To investigate the effects of acute and chronic sustained hypoxia in Aβ generation *in vivo*.

**Methods:**

2–3 month-old C57/Bl6J wild-type mice were exposed to either normoxia (21% O_2_) or hypoxia (9% O_2_) for either 4 to 72 h (acute) or 21–30 days (chronic sustained) in a hermetic chamber. Brain mRNA levels of Aβ-related genes were measured by quantitative real-time PCR, whereas levels of Bace1 protein, full length AβPP, and its C-terminal fragments (C99/C88 ratio) were measured by Western blot. In addition, 8 and 14-month-old APP/PS1 transgenic mice were subjected to 9% O_2_ for 21 days and levels of Aβ_40_, Aβ_42_, full length AβPP, and soluble AβPPα (sAβPPα) were measured by ELISA or WB.

**Results:**

Hypoxia (either acute or chronic sustained) did not impact the transcription of any of the Aβ-related genes in young wild-type mice. A significant reduction of Bace1 protein level was noted with acute hypoxia for 16 h but did not correlate with an increased level of full length AβPP or a decreased C99/C83 ratio. Chronic sustained hypoxia did not significantly alter the levels of Bace1, full length AβPP or the C99/C83 ratio. Last, chronic sustained hypoxia did not significantly change the levels of Aβ_40_, Aβ_42_, full length AβPP, or sAβPPα in either young or aged APP/PS1 mice.

**Discussion:**

Our results argue against a hypoxia-induced shift of AβPP proteolysis from the non-amyloidogenic to the amyloidogenic pathways. We discuss the possible methodological caveats of previous *in vivo* studies.

## Introduction

Alzheimer disease (AD) is the most common neurodegenerative disease and the most prevalent dementia. AD is defined neuropathologically by the presence of amyloid plaques and neurofibrillary tangles (NFTs) in sufficient number and extension within the cortex. While NFTs are intraneuronal somatodendritic aggregates of the hyperphosphorylated and misfolded microtubule-associated protein tau, amyloid plaques are extracellular deposits of amyloid-β (Aβ) peptide, which is released by the neurons to the interstitial space [1]. Aβ is a normal by-product of the transmembrane amyloid-β precursor protein (AβPP) after its sequential cleavage by the transmembrane aspartyl proteases β- and γ-secretases. Specifically, the cleavage of AβPP by the β-site AβPP cleaving enzyme 1 (Bace1) produces two fragments: soluble AβPPβ (sAβPPβ) and a 99 amino acid C-terminal fragment (βCTF or C99). The latter is next cleaved by γ-secretase to produce Aβ peptides of different lengths from 37 to 43 amino acids depending on the cleaving site. The γ-secretase is, in fact, a complex of four proteins: presenilin 1 or 2—which contains the catalytic proteolytic site—, Aph1 (with one of three isoforms A, B, or C), nicastrin, and Pen-2.

AβPP can be alternatively cleaved within the Aβ region by α-secretase enzymes, which give rise to soluble AβPPα (sAβPPα) and an 83 amino acid C-terminal fragment (αCTF or C83), preventing Aβ generation. The main α-secretases responsible for the non-amyloidogenic proteolysis of AβPP are the members of the A disintegrin and metalloprotease (ADAM) family Adam9, Adam10, and Adam17 (also called *tumor necrosis α converting enzyme* or Tace). A number of Aβ-degrading enzymes have been reported, among which the most relevant and well-studied are neprilysin and insulin-degrading enzyme (Ide). While Bace1 initiates the amyloidogenic processing of AβPP that leads to Aβ, Bace2 actually prevents Aβ generation and may degrade Aβ (reviewed in [2–4]).

The pathophysiological hypothesis of AD that has prevailed for over two decades regards Aβ accumulation as the trigger of a cascade of adverse events that ultimately leads to synaptic loss, tau aggregation into NFTs, neuron death, and dementia [5,6]. This process is accelerated by several known risk genetic polymorphisms, the strongest of which is the ε4 allele of the apolipoprotein E gene (*APOE*), but the acquired environmental factors upstream Aβ accumulation are less well established. Epidemiological studies have suggested that vascular risk factors in midlife may increase the risk of developing AD later in life. Population-based clinicopathologic studies have demonstrated that the most common pathological substrate of dementia in community-dwelling elderly people is actually mixed AD with cerebrovascular disease [7,8]. Recent epidemiological studies have specifically attributed an increased risk of dementia and AD to hypoxia-causing diseases such as chronic obstructive pulmonary disease (COPD) and obstructive sleep apnea (OSA) [9–12].

A growing body of literature has linked hypoxia with the accumulation of Aβ peptide through the regulation of Aβ-related genes [13–25]. Here, we investigated the effects of hypoxia on Aβ generation *in vivo*. Specifically, we tested the effects of acute hypoxia (AH) and chronic sustained hypoxia (CSH) with or without subsequent reoxygenation on the transcription of Aβ-related genes and AβPP processing in wild-type mice. We also investigated the effects of CSH on AβPP processing in APP/PS1 transgenic mice.

## Materials and Methods

### Animals

Heterozygous B6.Cg-Tg(APPswe;PSEN1ΔE9)85Dbo/J mice (APP/PS1 mice) were obtained from the Jackson Laboratory (stock number 005864) and control wild-type littermates were obtained by crossing them with C57/Bl6J mice. Mice were genotyped according to the vendor’s protocol. Mice were housed under controlled temperature (22°C) and humidity conditions in a 12 h light/dark cycle with *ad libitum* access to food and water. Housing and treatments were performed according to the animal care guidelines of European Community Council (86/60/EEC). The Animal Research Committee at the Hospitales Universitarios Virgen del Rocío approved all procedures. Experimental groups were homogenously distributed by sex. The number of mice used per group was kept to a minimum in accordance with the above guidelines.

### *In vivo* hypoxia treatment

Mice were exposed to a 9% O_2_ environment by using a hermetic chamber specially designed for hypoxia studies in animals (Coy Laboratory Products, Inc., Grass Lake, MI). This chamber is equipped with O_2_ and CO_2_ controllers and temperature and humidity monitoring [26–28]. A pump connected with the humidity and CO_2_ controllers mobilizes the air within the chamber and enables to regulate these parameters. O_2_ levels were monitored every other day and only ±0.5% differences were considered acceptable. CO_2_ within the chamber was kept at a minimum (< 0.1%) by filtering the air through a sodasorb (Grace) filter. Humidity was maintained below 70% at all times by filtering the air through a silica gel (Panreac) filter. Access into the chamber to feed the mice and clean their cages was achieved without altering the experimental conditions through glove ports and an airlock (transfer chamber). Light/dark, feeding, and cleaning cycles were kept uniform for all groups.

2-3-month-old wild-type mice were exposed to either acute hypoxia (AH, 9% O_2_, 4 to 72 h), with or without subsequent 24 h of reoxygenation, or chronic sustained hypoxia (CSH, 9% O_2_ uninterrupted for 21 or 30 days), with or without subsequent reoxygenation for 24 h. 8 and 14-month-old APP/PS1 mice were exposed to CSH (9% O_2_, 21 days) without reoxygenation. Littermate wild-type and APP/PS1 “normoxic” control mice of the same ages as above were also exposed to the same chamber for the same period of time but under a 21% O_2_ environment.

Animals were euthanized within the hypoxia chamber by administration of a lethal dose of anesthesia (sodium thiopental, thiobarbital). Brains were extracted within the hypoxia chamber, snap frozen in liquid nitrogen, and stored at –80°C for later use (qRT-PCR, WB, and ELISA).

### Quantitative RT-PCR

RNA was extracted from whole brains with a homogenizer (Omni TH) in the presence of TRIzol (Invitrogen). RNA samples (0.5 μg) were treated with RNase-free DNase (GE Healthcare) and retro-transcribed to cDNA using SuperScriptII reverse transcriptase (Invitrogen) in a final volume of 20 μL. Real time PCR was performed in an ABI Prism 7500 Sequence Detection System (Applied Biosystems) using SYBR-Green Master Mix (Applied Biosystems). A real time PCR reaction for *18S* mRNA was included in each 96-well plate to normalize results by RNA input amounts. Quantitation was performed using the ΔΔCt method. Primer sequences are listed in Table 1.

**Table 1:** Primers Used for Quantitative Real Time PCRs.

*mRNA*	Forward primer	Reverse primer
*App*	5’-CAAAGAGACATGCAGCGAGAAG-3’	5’-AGCATGCCATAGTCGTGCAA-3’
*Adam9*	5’-GTGCATATGGCGACTGTTGTAAA-3’	5’-ATGGAGCCTCCTGGAAGGAA-3’
*Adam10*	5’-GTTGCCGCCTCCTAAACCA-3’	5’-GGCGGTCTCCTCCTCTTTAAAG-3’
*Adam17/Tace*	5’-AACAACGACACCTGCTGCAATA-3’	5’-CTGCACACCCGGCTTCAG-3’
*Bace1*	5’-CAATCAGTCCTTCCGCATCAC-3’	5’-ACCGGCCGTAGGTATTGCT-3’
*Bace2*	5’-TTTGGTATCTCCTCTTCCACAAATG-3’	5’-CATCACGGTCGCACCAATC-3’
*Aph1a*	5’-CCTTCTACAGGAAGTGTTCCGTTT-3’	5’-TCTGCCTTCTTAAGGAGCTTGTAGT-3’
*Aph1b-c*	5’-GCGACTGTTGGCCTATGTTTCT-3’	5’-CACTCCACTCATGATTCCAAAGC-3’
*Psen1*	5’-CTCATGGCCCTGGTATTTATCAA-3’	5’-GAGCCATGCGGTCCATTC-3’
*Psen2*	5’-GCGAAACGTGTGATCATGCTAT-3’	5’-CACGATCATACACAGCGTGACA-3’
*Ncstn*	5’-CAAGCAGTGCTATCAAGATCACAA-3’	5’-CTTGGTGCAGAGCCATTCTG-3’
*Pen2*	5’-AGTTGAACCTGTGCCGGAAGT-3’	5’-AAGGCAGGAACGCAAATCC-3’
*Ide*	5’-GAGGTGAACGCTGTCGATTCA-3’	5’-GGCATCGTTCATCACATTCTTCT-3’
*Mme*	5’-ATTAAGTCTGTCCCTAGTGGCTAAGAA-3’	5’-GCAACACAGATACAGTTAGACCCTTTC-3’

*Mme* = Neprilysin; *Ncstn* = Nicastrin.

### Protein extraction

A hemibrain (wild-type mice) or hemi-cortex (APP/PS1 mice) was dissected and homogenized with a dounce grinder (Sigma) in a 4x w/v solution of phosphate-buffered saline (PBS) 1x containing protease inhibitor cocktail (Roche, 1:1,000) and phosphatase inhibitor (Sigma, 1:100). Total protein extracts were subjected to a multistep centrifugation to separate soluble from insoluble fractions. First, the total extracts were centrifuged at 600 g for 5 min at 4°C to pellet the nuclear fraction. Next, the resultant supernatants were ultracentrifuged at 100,000 g for 1 h at 4°C to separate PBS-insoluble (pellets) from PBS-soluble proteins (supernatants).

Nuclear and PBS-soluble fractions were stored at –80°C until use. The 100,000 g pellets were dissolved in a solution of 8.2 M guanidine HCl and 50 mM Tris-HCl to a final concentration of 5 M guanidine and incubated for 4 h at room temperature, with occasional inversion of the microcentrifuge tubes. These samples were stored at –20°C. Protein concentration in soluble and insoluble fractions was quantified using the RC-DC protein assay kit (Biorad), with bovine serum albumin (BSA) for the standard curve, following the manufacturer’s instructions.

### Aβ ELISA

Aβ_40_ and Aβ_42_ ELISA kits were purchased from Invitrogen. ELISA was performed on PBS-insoluble protein extracts following manufacturer’s instructions. Thawed 5 M guanidine HCl samples were diluted 1:50 in reaction buffer BSAT-DPBS containing PBS 1x, 5% bovine serum albumin, 0.03% Tween-20, and protease inhibitor (Invitrogen, 1:1,000). After a centrifugation step of 16,000 g for 20 min at 4°C, the supernatant was carefully removed and placed on ice until used for the assay.

### Vegf ELISA

Mouse vascular endothelial growth factor (Vegf) ELISA kit was purchased from R&D Systems (Quantikine, MMV00) and used according to manufacturer’s instructions.

### Western blots

Brain (wild-type mice) or cortical (APP/PS1 mice) protein extracts were subjected to SDS-PAGE using standard procedures except for the C-terminal fragments of AβPP, which required a Tris-tricine gel. AβPP and Bace1 were detected in the PBS-insoluble fraction, HIF1α in the nuclear fraction, and sAβPPα in the PBS-soluble fraction. Guanidine fractions were prepared for Western blot using 2-D Clean-Up kit (GE Healthcare Life Sciences).

The following primary antibodies were used: mouse monoclonal anti-β-actin (clone AC-15, Abcam, ab6276, 1:5,000), rabbit polyclonal anti-human AβPP C-terminal (751–770) (Millipore, #171610, 1:5,000), mouse monoclonal antibody anti-human sAβPPα (clone 2B3, TaKaRa, #11088, 1:500), rabbit monoclonal anti-Bace (clone D10E5, Cell signaling, #5606, 1:1,000), rabbit polyclonal anti-HIF1α C-terminal (Cayman, #10006421, 1:100), mouse monoclonal anti-pan-cadherin (clone CH-19, Abcam, ab6528, 1:1,000), rabbit polyclonal anti-Ribosomal Protein L26 (Sigma, R0655, 1:1,000), and mouse monoclonal anti-α-tubulin (clone B-5-1-2, Sigma, T5168, 1:4,000). HRP-conjugated secondary antibodies used were a sheep anti-mouse IgG antibody (GE Healthcare, NA931, 1:10,000) and a goat anti-rabbit IgG (ThermoFisher, #31460, 1:10,000). Signal was obtained with the Immun-Star^™^ WesternC^™^ Chemiluminescence Kit (Bio-Rad). The PVDF membrane was scanned on an ImageQuant scanner and the signal was quantified using ImageQuant software (GE Healthcare Life Sciences).

### Statistical analyses

Given the small size of the groups, only non-parametric statistical tests were used. Comparisons between two groups were performed with Mann-Whitney *U* test, whereas comparisons among three groups were done with Kruskal-Wallis ANOVA with Dunn’s multiple comparison test. Level of significance was set at *p* < 0.05. Data are expressed as mean ± s.e.m. Statistical analyses and graphs were performed in GraphPad Prism version 7.0 (GraphPad Inc., La Jolla, CA).

## Results

### Characterization of hypoxia treatment

**Fig 1:**
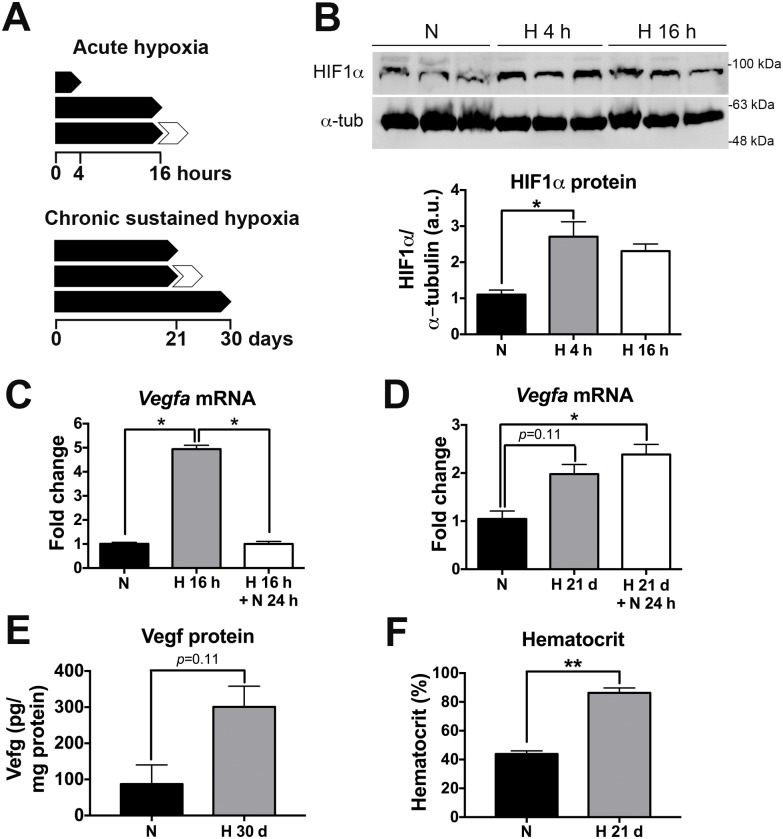
Characterization of hypoxia treatment protocols used in this study. (A) Schematic of acute (*left*) and chronic sustained (*right*) hypoxia treatment protocol used in this study. White arrowheads represent reoxygenation (21% O_2_) for 24 h. (B) *Left*, WB for HIF1α in brain extracts from 2–3 month-old wild-type mice subjected to AH (9% O_2_) for either 4 h or 16 h. *Right*, quantification of HIF1α WB. *p* < 0.05; Kruskal-Wallis ANOVA with Dunn’s multiple comparison test, *n* = 3 per group. (C) *Vegfa* mRNA levels measured by qRT-PCR in 2–3 month-old wild-type mice in normoxia and after AH (9% O_2_) for 16 h. Note the ~5-fold up-regulation of *Vegfa* expression caused by AH, which was reverted by 24 h reoxygenation. * *p* < 0.05; Kruskal-Wallis ANOVA with Dunn’s multiple comparison test, *n* = 4 per group. (D) *Vegfa* mRNA levels measured by qRT-PCR in 2–3 month-old wild-type mice in normoxia and after CSH (21 days, 9% O_2_), with and without reoxygenation (24 h, 21% O_2_). Note the ~2-fold up-regulation caused by CSH, which was not reverted by 24 h reoxygenation. Kruskal-Wallis ANOVA with Dunn’s multiple comparison test, *n* = 4 per group. (E) Vegf protein levels were measured by ELISA in 2–3 month-old wild-type mice subjected to either CSH (30 days, 9% O_2_) or normoxia (30 days, 21% O_2_ within the same chamber). A non-significant ~3-fold increase was observed in CSH compared to normoxia. Mann-Whitney *U* test, *n* = 4 per group. (F) Hematocrit of 14-month-old APP/PS1 mice subjected to CSH (21 days, 9% O_2_) or normoxia (21 days, 21% O_2_ within the same chamber). CSH was associated with a ~2-fold increase. *p* = 0.003; Mann-Whitney *U* test, *n* = 4 per group. Bars ± error bars represent mean ± s.e.m. HIF1α = hypoxia inducible factor 1 alpha; α-tub = alpha-tubulin; Vegf = vascular endothelial growth factor.

Fig 1A summarizes the main acute and chronic sustained hypoxia protocols used in this study. Fig 1B and 1C show the characterization of the effects of the acute hypoxia (AH, 9% O_2_) treatment. AH for 4 and 16 h stabilized the hypoxia inducible factor 1 alpha (HIF1α) within the brain of 2–3 month-old wild-type C57/Bl6J mice, compared to control littermates exposed to the same chamber for the same period of time but under normoxic conditions (21% O_2_) (Fig 1B). AH for 16 h caused an expected 5-fold up-regulation in the expression of the hypoxia-inducible gene *Vegfa* within the brain of these 2–3 month-old wild-type C57/Bl6J mice, which was rapidly corrected following 24 h of reoxygenation (21% O_2_) (*p* < 0.05 for the comparison with AH without reoxygenation) (Fig 1C).

Fig 1D–1F show the characterization of the effects of chronic sustained hypoxia (CSH, 9% O_2_) treatment. CSH for 21 days led to a ~2-fold increase of *Vegfa* mRNA levels in the brain of 2–3 month-old wild-type C57/Bl6J mice relative to control littermates exposed to the same chamber for the same period of time but at a 21% O_2_ level (normoxia), although this change did not reach statistical significance. Reoxygenation for 24 h after CSH was not sufficient to normalize brain *Vegfa* mRNA levels, which remained similarly high or slightly higher (≈2.3-fold, *p* < 0.05) (Fig 1D). Brain Vegf protein levels measured by ELISA also revealed a statistically non-significant ~3-fold increase under CSH conditions (30 days, 9% O_2_) (Fig 1E). Compared with normoxic conditions, CSH for 21 days essentially doubled the hematocrit of 14-month-old APP/PS1 mice (normoxia: 43.9±2.1%, CSH: 86.2±3.4%, *p* = 0.003) (Fig 1F), confirming that the physiological response to hypoxia is well preserved in these transgenic mice.

### Acute hypoxia does not regulate the transcription of Aβ-related genes in wild-type mice

**Fig 2:**
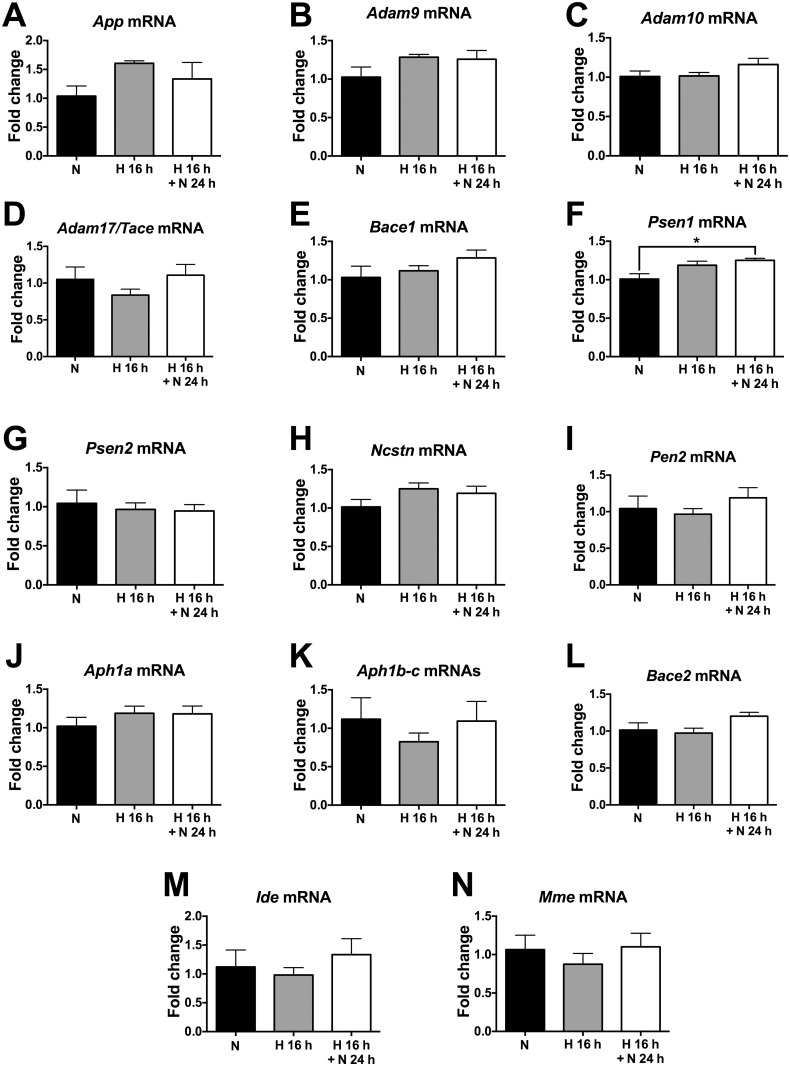
Acute hypoxia does not impact the transcription of Aβ-related genes in wild-type mice. Relative levels of the mRNAs encoding for AβPP (A), the α-secretases Adam9 (B) Adam10 (C), and Adam17/Tace (D), the β-secretase Bace1 (E), all the components of γ-secretase complex [presenilin-1 (F), presenilin-2 (G), nicastrin (H), pen-2 (I), Aph1a (J), and Aph1b-c (K)], and the Aβ-degrading enzymes β-secretase 2 or Bace2 (L), insulin-degrading enzyme or Ide (M), and neprilysin (N) were estimated in the brain of 2–3 month-old wild-type mice subjected to either 16 h of hypoxia (9% O_2_), with or without 24 h of reoxygenation, or 16 h of normoxia (21% O_2_) within the same chamber. *18S* mRNA was used as housekeeping control. * *p* < 0.05, Kruskal-Wallis ANOVA with Dunn’s multiple comparison test, *n* = 4 per group. Bars ± error bars represent mean ± s.e.m. *Mme* = Neprilysin; *Ncstn* = Nicastrin.

We next measured the brain levels of mRNAs encoding AβPP and the relevant Aβ-related enzymes in 2–3 month-old wild-type C57/Bl6J mice subjected to AH (16 h, 9% O_2_) with or without reoxygenation (24 h, 21% O_2_) using qRT-PCR. We found no statistically significant regulation of the transcription of any of the Aβ-related genes under these hypoxia conditions compared to normoxia (Fig 2A–2N). Only *Psen1* mRNA was significantly increased by 16 h of AH followed by 24 h of reoxygenation relative to normoxia, but this transcriptional up-regulation of the *Psen1* gene was quantitatively mild (≈1.25-fold) (Fig 2F).

### Chronic sustained hypoxia does not regulate the transcription of Aβ-related genes in wild-type mice

**Fig 3:**
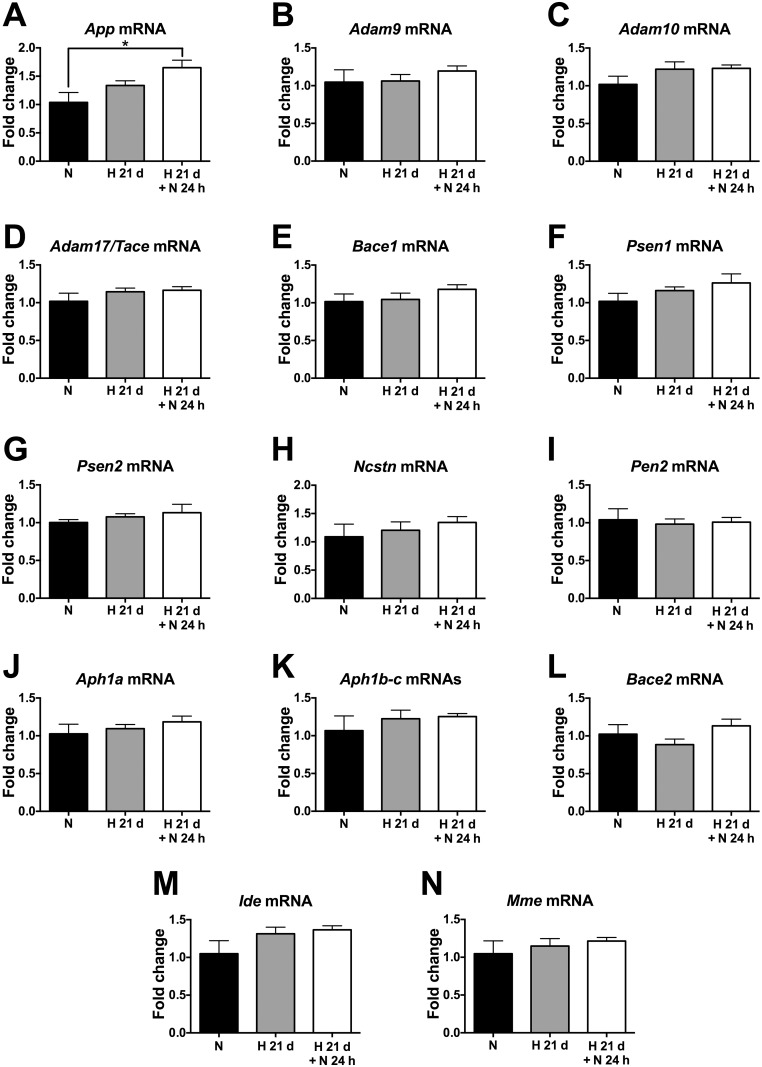
Chronic sustained hypoxia does not impact the transcription of Aβ-related genes in wild-type mice. Relative levels of the mRNAs encoding for AβPP (A), the α-secretases Adam9 (B) Adam10 (C), and Adam17/Tace (D), the β-secretase Bace1 (E), all the components of γ-secretase complex [presenilin-1 (F), presenilin-2 (G), nicastrin (H), pen-2 (I), Aph1a (J), and Aph1b-c (K)], the Aβ-degrading enzymes β-secretase 2 or Bace2 (L), insulin-degrading enzyme or Ide (M), and neprilysin (N) were estimated in the brain of 2–3 month-old wild-type mice subjected to either 21 days of hypoxia (9% O_2_), followed or not by 24 h of reoxygenation (21% O_2_), or 21 days of normoxia (21% O_2_) within the same chamber. *18S* mRNA was used as housekeeping control. * *p* < 0.05, Kruskal-Wallis ANOVA with Dunn’s multiple comparison test, *n* = 4 for the normoxia group and *n* = 5 for the two hypoxia groups. Bars ± error bars represent mean ± s.e.m. *Mme* = Neprilysin; *Ncstn* = Nicastrin.

Similarly, we measured the brain levels of mRNAs encoding AβPP and the relevant Aβ-related enzymes in 2–3 month-old wild-type C57/Bl6J mice subjected to CSH (21 days, 9% O_2_), followed or not by reoxygenation (24 h, 21% O_2_), using qRT-PCR. None of the above mentioned Aβ-related genes exhibited a statistically significant regulation of their transcription by CSH (Fig 3A–3N). Only *App* mRNA level was significantly changed by CSH followed by 24 h reoxygenation compared with normoxia (≈1.6-fold increase, *p* < 0.05) (Fig 3A).

### Neither AH nor CSH change AβPP processing in wild-type mice

**Fig 4:**
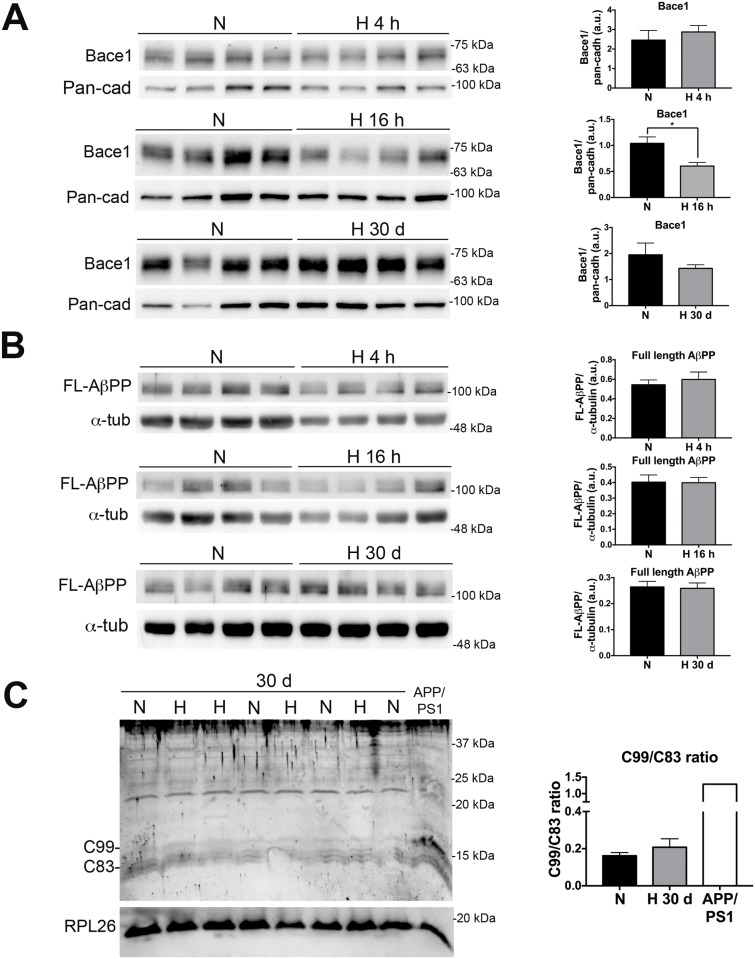
Neither acute nor chronic sustained hypoxia impact AβPP processing. (A) Bace1 protein levels were measured by WB in the brain of 2–3 month-old wild-type mice subjected to either AH (9% O_2_, 4 or 16 h) or CSH (9% O_2_, 30 days), or to normoxia (21% O_2_) for the corresponding period of time within the same chamber. Right bar graphs depict the quantification of WBs using pan-cadherin as loading control. Note that, compared to normoxia, Bace1 levels were significantly reduced after 16 h of hypoxia; * *p* < 0.05, Mann-Whitney *U* test, *n* = 4 per group. However, no change was observed after 4 h of hypoxia, and its decrease did not reach statistical significance after CSH. (B) Levels of full length AβPP were measured by WB. Right bar graphs show the quantification of WBs using α-tubulin as loading control. No significant change was observed with any hypoxia protocol (*n* = 4 per group). (C) The C99 and C83 fragments of AβPP were measured by WB and the C99/C83 ratio was calculated. No significant change was observed by CSH. The rightmost lane of the Tris-tricine gel was loaded with a protein extract from an APP/PS1 transgenic mouse as positive control. The membrane was reprobed with an anti-RPL26 antibody to demonstrate equal protein load in all gel lanes. Bars ± error bars represent mean ± s.e.m. FL-AβPP = full length AβPP; Pan-cad = pan-cadherin; RPL26 = Ribosomal Protein L26; α-tub = alpha-tubulin.

We next tested whether AH or CSH alter AβPP processing. β-site AβPP cleaving enzyme 1 (Bace1), the limiting enzyme in the amyloidogenic pathway of AβPP processing, has been proposed as an injury-sensor enzyme whose levels increase in the setting of acute insults such as ischemia and energy deprivation [29,30]. In fact, the results of several prior *in vitro* and *in vivo* hypoxia studies support this hypothesis [15,17,19,20,25]. Therefore, we measured the levels of Bace1 by WB and compared them between normoxia and AH or CSH (Fig 4A). Bace1 levels remained unchanged after AH for 4 h. Surprisingly however, Bace1 levels not only did not increase but significantly decreased after AH for 16 h and also appeared to be reduced after CSH for 30 days, although not significantly.

Unfortunately, endogenous murine brain Aβ levels were too low to be measured reliably in these 2–3 month-old wild-type mice. Therefore, we evaluated the processing of AβPP in wild-type mice by measuring the levels of full length AβPP and the ratio of its by-products C99 and C83 (C99/C83 ratio) by WB. Full length AβPP levels did not differ between normoxic and hypoxic mice exposed to either AH or CSH (Fig 4B). Similarly, the C99/C83 ratio did not change after AH for 32 or 72 h (data not shown) or CSH (Fig 4C).

### Chronic sustained hypoxia does not alter Aβ, full length AβPP, or sAβPPα levels in APP/PS1 mice

**Fig 5:**
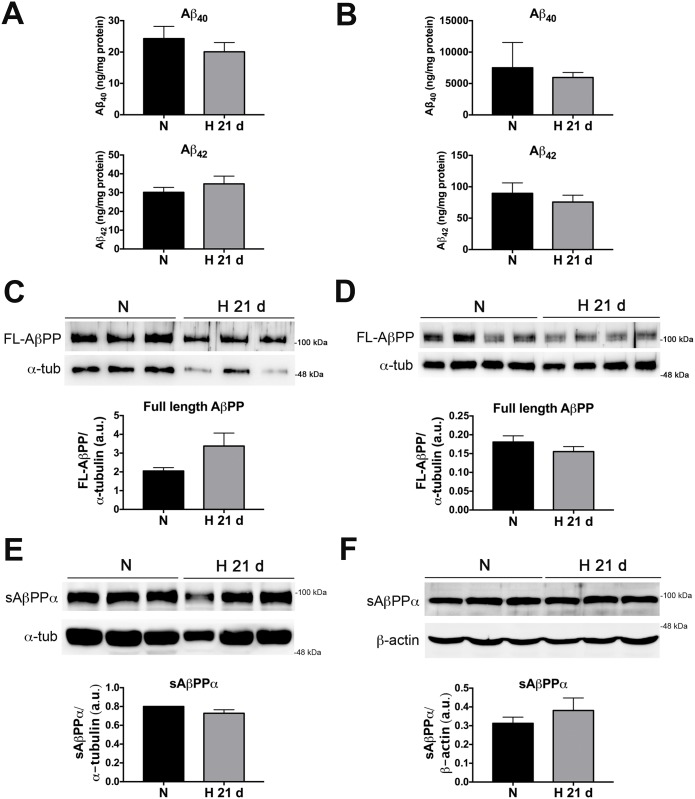
Chronic sustained hypoxia does not significantly alter the brain levels of Aβ_40_, Aβ_42_, full length AβPP, and sAβPPα in APP/PS1 mice. Levels of Aβ_40_ and Aβ_42_ were measured by ELISA (A-B) and levels of full length AβPP (C-D) and sAβPPα (E-F) were determined by WB in protein extracts from the cortex of 8-month-old (A, C, E) and 14-month-old (B, D, F) APP/PS1 mice exposed to CSH (9% O_2_, 21 days) or normoxia (21% O_2_, 21 days within the same chamber). Mann-Whitney *U* test, *n* = 3–4 per group for Aβ ELISAs and AβPP WBs and *n* = 3 per group for sAβPPα WBs. Bars ± error bars represent mean ± s.e.m. FL-AβPP = full length AβPP; sAβPPα = soluble AβPP alpha; α-tub = alpha-tubulin.

Last, we asked whether our CSH paradigm could increase Aβ levels and/or impact AβPP processing in the presence of Aβ deposits. To answer this question we measured the cortical levels of Aβ_40_ and Aβ_42_ by ELISA and the levels of full length AβPP and sAβPPα by WB in 8 and 14-month-old APP/PS1 mice exposed to either CSH (21 days, 9% O_2_) or normoxia (21 days, 21% O_2_ within the same chamber). These ages are representative of low and high Aβ burden, respectively [31]. CSH did not significantly change the levels of Aβ_40_ or Aβ_42_ as compared to normoxia in either young (Fig 5A) or old (Fig 5B) APP/PS1 mice. Accordingly, no changes were observed in the levels of full length AβPP at any age analyzed (Fig 5C and 5D). Similarly, WB for sAβPPα revealed no significant difference in its levels between the conditions of normoxia and CSH in either 8 (Fig 5E) or 14-month-old (Fig 5F) APP/PS1 mice.

## Discussion

We present several lines of evidence arguing against a hypoxia-induced shift of AβPP proteolysis from the non-amyloidogenic to the amyloidogenic pathways in both wild-type and AD mice. First, exposure of young wild-type mice to either AH or CSH did not significantly change the transcription of any of the Aβ-related genes. Second, Bace1 protein level was not only not increased by hypoxia, but decreased by both AH for 16 h and, to a lesser extent, CSH for 30 days, in young wild-type mice. Nonetheless, AβPP processing—as indicated by WBs for full length AβPP and C99/83 ratio—was not altered by either hypoxia protocol in these young wild-type mice. Last, Aβ_40_ and Aβ_42_, full length AβPP, and sAβPPα levels were not significantly changed by exposure to CSH in either young (low Aβ burden) or aged (high Aβ burden) APP/PS1 mice.

Our results are at odds with a number of prior *in vitro* and *in vivo* studies (Table 2), which support a pro-amyloidogenic effect of hypoxia on Aβ metabolism through a variety of mechanisms, including the up-regulation of AβPP itself [13,20,23,25], the up-regulation of its amyloidogenic enzymes such as Bace1 [15,17,19,20,25], presenilin-1 [14,20], presenilin-2 [20], Aph1a [16,18,25], nicastrin [25], and pen-2 [25], and the down-regulation of the Aβ-degrading enzyme neprilysin [23–25]. As the main transcription factor that orchestrates the physiologic response to hypoxia [32], the presence of HIF1 regulatory elements (HRE) in the 5’ UTR of these Aβ-related genes is, indeed, a very appealing observation invoked by some of these studies to support their role as HIF target genes.

**Table 2:** Literature Review on Regulation of Aβ Metabolism by Hypoxia.

**Results**	**Behavior**	NA	NA	NA	NA	↓ MWM	NA	NA	NA	NA	NA	NA	= MWM	NA	↓ MWM,↓ TST = OF	↓ MWM	NA	↓ MWM
	**Synapses**	NA	NA	NA	NA	NA	NA	NA	NA	NA	NA	NA	NA	NA	*NA*	↓ syn,↓ EM	NA	↓ syn,↓ EM
	**Tau**	↑ tau	NA	NA	NA	NA	NA	NA	NA	NA	NA	NA	NA	NA	↑ p-tau, = tau	↑ p-tau	NA	= NFT number,↑ p-tau/tau ratio (at 4 mo)
	**Neprilysin**	NA	NA	NA	NA	NA	NA	NA	NA	NA	NA	NA	NA	NA	NA	↓ Neprilysin	↓ *Mme*,↓ Neprilysin level & activity	↓ Neprilysin
	**Aβ**	↑ Aβ	↑ Aβ	↑ Aβ_40_,↑ Aβ_42_	↑ Aβ_40_,↑ Aβ_42_	↑ Aβ_40_,↑ Aβ_42_,↑ plaque number	NA	↑ Aβ_40_,↑ Aβ_42_	↑ Aβ_42_	↑ soluble & FA-Aβ_42_,↑ plaque area & number	NA	NA	↑ Aβ_42_,↑ intraneuronal Aβ, = Aβ_40_	↑ Aβ_42_, = Aβ_40_	↑ Aβ_42_,↑ plaque area & number	↑ soluble & FA hu Aβ_42_ & Aβ_40_,↑ soluble mu Aβ_42_ & Aβ_40_,↑ plaque area & number	NA	↑ soluble & FA Aβ_42_/Aβ_40_ ratio,↑ plaque area & number
	**Y-secretase**	NA	↑ Presenilin-1	NA	NA	NA	↑ *Aph1a &* Aph1a, = Presenilin-1, = Nicastrin, = Pen2	= Presenilin 1	↑ Aph-1a	↑ Aph-1a	NA	↑ *Psen1*,↑ *Psen2*	NA	NA	NA	NA	NA	↑ Aph1a,↑ Nicastrin,↑ Pen2, = Presenilin-1
	**BACE**	NA	NA	↑ *Bace1* & Bace1	NA	↑ *Bace1* (in wt)	NA	↑ *Bace1 &* Bace1	NA	NA	↑ *Bace1* & Bace1	↑ *Bace1*	= Bace1	NA	NA	= Bace1	NA	↑ Bace1 (in wt)
	**APP**	↑ AβPP	NA	↑ C99	↑ C99	↑ C99	↑ sAβPPαα, = AβPP,↓ AβPP-CTFs	↑ C99, = AβPP	↓ HA-C99	↑ C99/C83 ratio	NA	↑ *Appa*,↑ *Appb*	= AβPP	NA	NA	↑ AβPP	NA	↑ AβPP, = C99/C83 ratio
**CO**_**2**_ **level**		5%	5%	5%	5%	NA	5%	NA	5%	NA	5%	NA	<0.03%	5%	NA	NA	NA	NA
**Hypoxia duration**		4 & 8 h followed by 20%O_2_ for 24 or 48 h	24 h	12 & 24 h	12 h	16 h/day for 1 mo	2, 4, 8, 12 & 20 h	2, 4 & 8 h	4 h	Once daily for 60 d	1, 3, 6, 12, 24, 48 & 72 h	Embryos: from 6 hpf to 24 or 48 hpf stage,Adults: 3 h	5% vs 21% every 10 min for 8 h per day during 8 weeks	1% 10 min vs 21% 20 min for 8 cycles	Once daily for 60 d	6 h/day for days 7 to 20 of gestation followed by normoxia up to age 3, 6 & 9 mo	24 h	6 h/day for 30 d followed by up to 5 mo normoxia prior to sacrifice
**Hypoxia level**		NA	2.5% O_2_	2% O_2_	2% O_2_	8% O_2_	NA	1% O_2_	NA	NA, until “first gasping breath”	3% O_2_	Embryos: ≈10% of controls,Adults: ≈17% of controls	5% O_2_	1% O_2_	NA, until “first gasping breath”	11.1% O_2_	1% O_2_	11.1% O_2_
**Hypoxia method**		Sealed but “not 100% leak-proof” chamber	Incubator	Incubator	Incubator	Semisealable hypoxia chamber	1 mM NiCl_2_	Incubator	1 mM NiCl_2_	Sealed 125 mL jar with fresh air	Incubator	Bubbling N2 to the medium	Hypoxia chamber	Incubator	Sealed 125 mL jar with fresh air	Hypobaric chamber	Incubator	Hypobaric chamber
**Model**		Rat cortical neuron primary culture	Rat cortical astrocyte primary culture	SH-SYS5-APP_swe_ cells	HEK-APP_695wt_	APP23 mice (8 mo, M:F 1:1)	HeLa-APP_swe_ cells	N2a-APP_695wt_ cells	SH-SYS5-C99 cells	APP_swe_/PS1_A246E_ mice (9 mo, F)	SK-N-BE neuroblastoma cells	Zebra fish embryos & adults	3xTg mice (6 mo, M)	SH-SYS5-APP_wt_ cells	APP_swe_/PS1_dE9_ mice (6 mo)	APP_swe_/PS1_A246E_ pregnant mice	NB7 (SJ-N-CG) neuroblastoma cells	APP_swe_/PS1_dE9_ mice (3 mo)
**Author / year**		Chen et al. 2003	Smith et al. 2004	Sun et al. 2006			Wang et al. 2006	Zhang et al. 2007	Li et al. 2009		Guglielmotto et al 2009	Moussavi Nik et al. 2012	Shiota et al. 2013		Gao et al. 2013	Zhang et al 2013	Kerridge et al 2015	Liu et al. 2016

Results of a search in the US National Library of Medicine of the National Institutes of Health (http://www.ncbi.nlm.nih.gov/pubmed/) using the combination of keywords “hypoxia AND Alzheimer”. Both *in vitro* and *in vivo* studies were included. *In vitro* studies used either exposure to a low O_2_ level within the cell incubator or treatment with hypoxia mimics (i.e. NiCl_2_ or DMOG), and either cell lines stably expressing an AβPP construct, (i.e. the 695 amino acid wild-type form or the Swedish mutation) or primary rat cortical cultures, both neuronal and astrocytic. Note: Articles were excluded if: 1) they exclusively described the effects of hypoxia on tau phosphorylation/pathology or some other aspect of AD pathophysiology (i.e. mitochondrial dysfunction) without addressing its effects on Aβ; 2) they used a paradigm other than pure hypoxia (i.e. ischemia, hypocapnia, oxygen and glucose deprivation, oxidative stress), and 3) they were written in a language different from English.

Abbreviations: ↓: significant decrease; ↑: significant increase; =: no significant change; d: days; EM: electron microscopy; F: female; FA: formic acid; h: hours; hu: human; M: male; *Mme* = neprilysin mRNA; mo: month; mu: murine; MWM: Morris water maze (↓ indicates worse performance); NA: not available; NFT: neurofibrillary tangle; OF: open field; syn: synaptophysin; TST: tail suspension test (↓ indicates worse performance). Note: mRNAs are expressed in *Italics*, whereas proteins are Capitalized.

Methodological differences may account for the discrepancy between present and previous results. First, the hypoxia paradigm used by other authors may not have been adequate. We have previously validated our hypoxia protocol in this chamber and showed that the resulting changes in the blood gasometry consist of a pure hypoxemia (i.e. reduced O_2_ saturation and *Pa*O_2_) without hypercapnia or acidosis [28]. Here we provide further evidence of the systemic and brain effects of our hypoxia protocols. AH induced a significant stabilization of the HIF1α protein and an up-regulation of the HIF1α-target gene *Vegfa*. CSH induced a notable up-regulation of both *Vegfa* mRNA and Vegf protein in wild-type mice and a marked polycythemia (i.e. increased hematocrit) in APP/PS1 mice. Moreover, we have previously shown that the oxygen level employed here (9%) is well tolerated by mice and does not provoke neuronal death [28]. By contrast, Li *et al*. [18] and Gao *et al*. [22] enclosed the mice in a 125 mL sealed jar “until last breath gasping” to cause hypoxia, a paradigm in which the levels of O_2_ and CO_2_ cannot be controlled. Liu *et al*. [25] and Zhang *et al*. [23] used a hypobaric chamber instead of a hypoxia chamber to cause hypoxia mimicking a high altitude scenario. The CO_2_ levels in these experiments and the possibility of off-target effects such as vasogenic edema from a hypobaric environment simulating high altitude illness were not reported [33,34]. Thus, these conditions may not faithfully represent the hypoxemia associated with human diseases such as COPD [35] and OSA [36] nor the local brain hypoxia occurring during a stroke [32].

Second, other researchers used a protocol of chronic intermittent hypoxia—from minutes to hours on a daily basis for up to 1 or 2 months—but attributed their findings to the repeated transient hypoxia rather than to the reoxygenation following each period of hypoxia [15,18,21–23,25], We observed that reoxygenation did cause a statistically significant increase in the levels of *Psen1* and *App* mRNAs in young wild-type mice after AH and CSH, respectively, but the magnitude of these changes was so small that their pathogenic significance remains uncertain. It should be noted that we only exposed the wild-type mice to one cycle of reoxygenation and cannot rule out that repetitive cycles of hypoxia/reoxygenation could lead to a more dramatic transcriptional regulation of these genes. Also noteworthy, to reproduce the intermittent brief (seconds to minutes) hypoxia that occurs in OSA, a different hypoxia chamber able to provide both drastic and rapid changes in FiO_2_ would be needed.

Last, the validity of the normoxia control group used in prior studies is also questionable. Because environmental enrichment is known to reduce Aβ levels [37] and chronic stress is known to increase Aβ levels [38–40], we placed the cages of control mice within the same hypoxia chamber for the same period of time but under normoxic conditions (21% O_2_). Thus, unlike prior studies, we neutralized potential changes on both the transcription of Aβ-related genes and the levels of Aβ peptide that could be attributable to the exposure of the mice to a new and/or potentially stressful environment.

## Conclusions

In summary, our results argue against a hypoxia-induced shift of AβPP processing leading to an increased Aβ generation. The possibility remains that reoxygenation after hypoxia (rather than hypoxia itself) is associated with an enhanced amyloidogenic processing of AβPP. Also, the potential indirect effects of hypoxia on Aβ metabolism, for example mediated through Vegf [41,42], were not investigated here. More studies are needed to elucidate the molecular mechanism(s) that underlie(s) the epidemiological evidence linking hypoxia and cerebrovascular ischemic disease with Alzheimer disease.
