# Correction: Acute and Chronic Sustained Hypoxia Do Not Substantially Regulate Amyloid-β Peptide Generation *In Vivo*

**DOI:** 10.1371/journal.pone.0181510

**Published:** 2017-07-26

**Authors:** The PLOS ONE Staff

There are several errors throughout the paper. The publisher apologizes for these errors. The figure legend for Fig 1 is incorrect. Please see the Fig 1 and the correct figure legend below.

**Fig 1:**
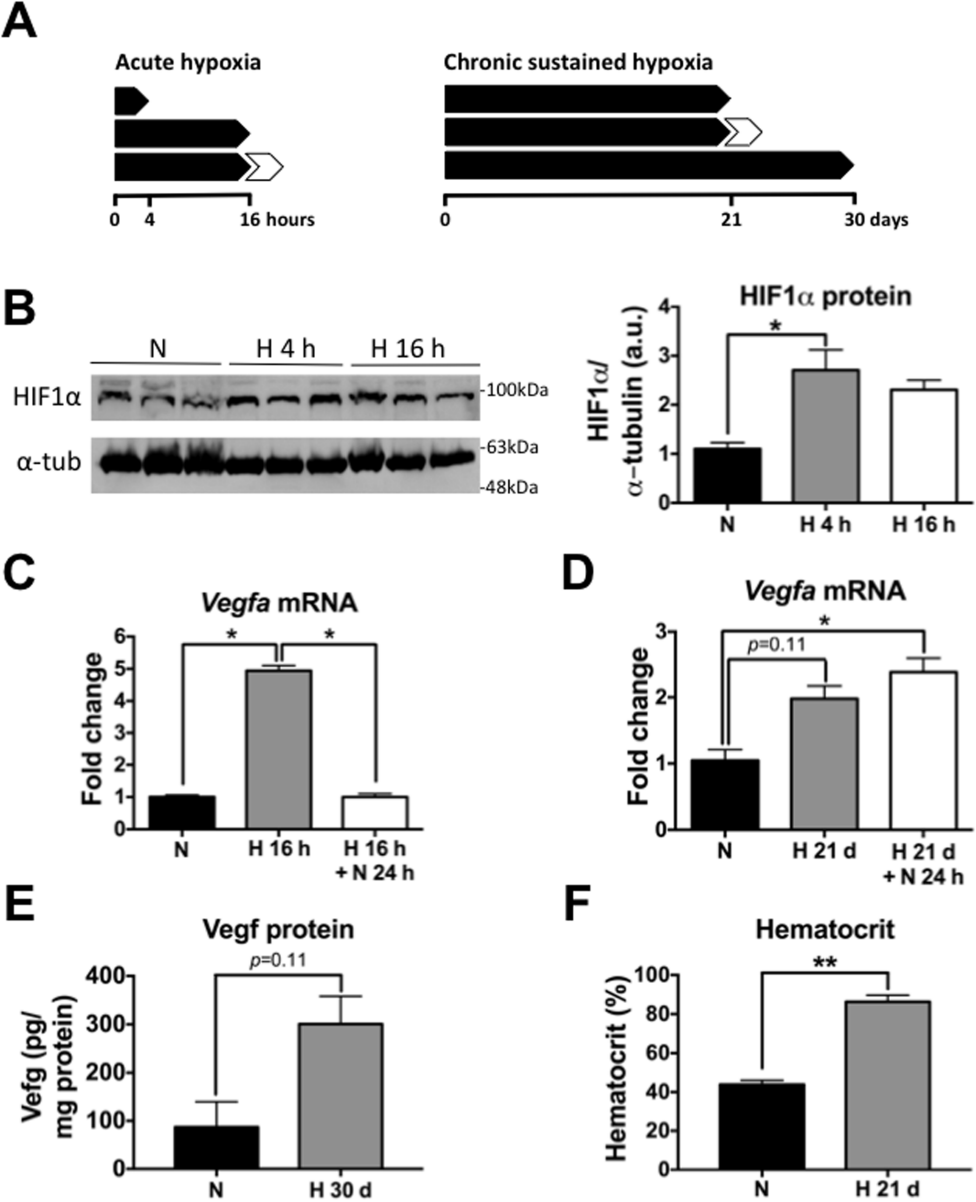
Characterization of hypoxia treatment protocols used in this study. (A) Schematic of acute (*top*) and chronic sustained (*bottom*) hypoxia treatment protocol used in this study. White arrowheads represent reoxygenation (21% O_2_) for 24 h. (B) *Top*, WB for HIF1α in brain extracts from 2–3 month-old wild-type mice subjected to AH (9% O_2_) for either 4 h or 16 h. *Bottom*, quantification of HIF1α WB. *p* < 0.05; Kruskal-Wallis ANOVA with Dunn’s multiple comparison test, *n* = 3 per group. (C) *Vegfa* mRNA levels measured by qRT-PCR in 2–3 month-old wild-type mice in normoxia and after AH (9% O_2_) for 16 h. Note the ~5-fold up-regulation of *Vegfa* expression caused by AH, which was reverted by 24 h reoxygenation. * *p* < 0.05; Kruskal-Wallis ANOVA with Dunn’s multiple comparison test, *n* = 4 per group. (D) *Vegfa* mRNA levels measured by qRT-PCR in 2–3 month-old wild-type mice in normoxia and after CSH (21 days, 9% O_2_), with and without reoxygenation (24 h, 21% O_2_). Note the ~2-fold up-regulation caused by CSH, which was not reverted by 24 h reoxygenation.* *p* < 0.05; Kruskal-Wallis ANOVA with Dunn’s multiple comparison test, *n* = 4 per group. (E) Vegf protein levels were measured by ELISA in 2–3 month-old wild-type mice subjected to either CSH (30 days, 9% O_2_) or normoxia (30 days, 21% O_2_ within the same chamber). A non-significant ~3-fold increase was observed in CSH compared to normoxia. Mann-Whitney *U* test, *n* = 4 per group. (F) Hematocrit of 14-month-old APP/PS1 mice subjected to CSH (21 days, 9% O_2_) or normoxia (21 days, 21% O_2_ within the same chamber). CSH was associated with a ~2-fold increase. *p* = 0.003; Mann-Whitney *U* test, *n* = 4 per group. Bars ± error bars represent mean ± s.e.m. HIF1α = hypoxia inducible factor 1 alpha; α-tub = alpha-tubulin; Vegf = vascular endothelial growth factor.

The Table 2 legend is incorrectly incorporated into the body of the manuscript. Additionally, the order of rows for Table 2 is incorrect. The correct order of columns from left to right should be: Author / year, Model, Hypoxia method, Hypoxia level, Hypoxia duration, CO_2_ level, Results (APP, BACE, γ-secretase, Aβ, Neprilysin, Tau, Synapses, Behavior). Please see the correct Table 2 and Table 2 legend below.

**Table 2:** Literature review on regulation of Aβ Metabolism by hypoxia. Results of a search in the US National Library of Medicine of the National Institutes of Health (http://www.ncbi.nlm.nih.gov/pubmed/) using the combination of keywords “hypoxia AND Alzheimer”. Both *in vitro* and *in vivo* studies were included. *In vitro* studies used either exposure to a low O_2_ level within the cell incubator or treatment with hypoxia mimics (i.e. NiCl_2_ or DMOG), and either cell lines stably expressing an AβPP construct, (i.e. the 695 amino acid wild-type form or the Swedish mutation) or primary rat cortical cultures, both neuronal and astrocytic. Note: Articles were excluded if: 1) they exclusively described the effects of hypoxia on tau phosphorylation/pathology or some other aspect of AD pathophysiology (i.e. mitochondrial dysfunction) without addressing its effects on Aβ; 2) they used a paradigm other than pure hypoxia (i.e. ischemia, hypocapnia, oxygen and glucose deprivation, oxidative stress), and 3) they were written in a language different from English. Abbreviations: ↓: significant decrease; ↑: significant increase; =: no significant change; d: days; EM: electron microscopy; F: female; FA: formic acid; h: hours; hu: human; M: male; *Mme* = neprilysin mRNA; mo: month; mu: murine; MWM: Morris water maze (↓ indicates worse performance); NA: not available; NFT: neurofibrillary tangle; OF: open field; syn: synaptophysin; TST: tail suspension test (↓ indicates worse performance). Note: mRNAs are expressed in *Italics*, whereas proteins are Capitalized.

Author / year	Model	Hypoxia method	Hypoxia level	Hypoxia duration	CO_2_ level	RESULTS							
						APP	BACE	Y-secretase	Aβ	Neprilysin	Tau	Synapses	Behavior
Chen et al. 2003	Rat cortical neuron primary culture	Sealed but “not 100% leak-proof” chamber	NA	4 & 8 h followed by 20%O_2_ for 24 or 48 h	5%	↑ AβPP	NA	NA	↑ Aβ	NA	↑ tau	NA	NA
Smith et al. 2004	Rat cortical astrocyte primary culture	Incubator	2.5% O_2_	24 h	5%	NA	NA	↑ Presenilin-1	↑ Aβ	NA	NA	NA	NA
Sun et al. 2006	SH-SYS5-APP_swe_ cells	Incubator	2% O_2_	12 & 24 h	5%	↑ C99	↑ *Bace1* & Bace1	NA	↑ Aβ_40_ ↑ Aβ_42_	NA	NA	NA	NA
	HEK-APP_695wt_	Incubator	2% O_2_	12 h	5%	↑ C99	NA	NA	↑ Aβ_40_ ↑ Aβ_42_	NA	NA	NA	NA
	APP23 mice (8 mo, M:F 1:1)	Semisealable hypoxia chamber	8% O_2_	16 h/day for 1 mo	NA	↑ C99	↑ *Bace1* (in wt)	NA	↑ Aβ_40_ ↑ Aβ_42_ ↑ plaque number	NA	NA	NA	↓ MWM
Wang et al. 2006	HeLa-APP_swe_ cells	1 mM NiCl_2_	NA	2, 4, 8, 12 & 20 h	5%	↑ sAβPPα = AβPP ↓ AβPP-CTFs	NA	↑ *Aph1a &* Aph1a = Presenilin-1 = Nicastrin = Pen2	NA	NA	NA	NA	NA
Zhang et al. 2007	N2a-APP_695wt_ cells	Incubator	1% O_2_	2, 4 & 8 h	NA	↑ C99 = AβPP	↑ *Bace1 &* Bace1	= Presenilin 1	↑ Aβ_40_ ↑ Aβ_42_	NA	NA	NA	NA
Li et al. 2009	SH-SYS5-C99 cells	1 mM NiCl_2_	NA	4 h	5%	↓ HA-C99	NA	↑ Aph-1a	↑ Aβ_42_	NA	NA	NA	NA
	APP_swe_/PS1_A246E_ mice (9 mo, F)	Sealed 125 mL jar with fresh air	NA, until “first gasping breath”	Once daily for 60 d	NA	↑ C99/C83 ratio	NA	↑ Aph-1a	↑ soluble & FA-Aβ_42_ ↑ plaque area & number	NA	NA	NA	NA
Guglielmotto et al 2009	SK-N-BE neuroblastoma cells	Incubator	3% O_2_	1, 3, 6, 12, 24, 48 & 72 h	5%	NA	↑ *Bace1* & Bace1	NA	NA	NA	NA	NA	NA
Moussavi Nik et al. 2012	Zebra fish embryos & adults	Bubbling N2 to the medium	Embryos: ≈10% of controls Adults: ≈17% of controls	Embryos: from 6 hpf to 24 or 48 hpf stage Adults: 3 h	NA	↑ *Appa* ↑ *Appb*	↑ *Bace1*	↑ *Psen1* ↑ *Psen2*	NA	NA	NA	NA	NA
Shiota et al. 2013	SH-SYS5-APP_wt_ cells	Incubator	1% O_2_	1% 10 min vs 21% 20 min for 8 cycles	5%	NA	NA	NA	↑ Aβ_42_ = Aβ_40_	NA	NA	NA	NA
	3xTg mice (6 mo, M)	Hypoxia chamber	5% O_2_	5% vs 21% every 10 min for 8 h per day during 8 weeks	<0.03%	= AβPP	= Bace1	NA	↑ Aβ_42_ ↑ intraneuronal Aβ = Aβ_40_	NA	NA	NA	= MWM
Gao et al. 2013	APP_swe_/PS1_dE9_ mice (6 mo)	Sealed 125 mL jar with fresh air	NA, until “first gasping breath”	Once daily for 60 d	NA	NA	NA	NA	↑ Aβ_42_ ↑ plaque area & number	NA	↑ p-tau = tau	NA	↓ MWM ↓ TST = OF
Zhang et al 2013	APP_swe_/PS1_A246E_ pregnant mice	Hypobaric chamber	11.1% O_2_	6 h/day for days 7 to 20 of gestation followed by normoxia up to age 3, 6 & 9 mo	NA	↑ AβPP	= Bace1	NA	↑ soluble & FA hu Aβ_42_ & Aβ_40_ ↑ soluble mu Aβ_42_ & Aβ_40_ ↑ plaque area & number	↓ Neprilysin	↑ p-tau	↓ syn ↓ EM	↓ MWM
Kerridge et al 2015	NB7 (SJ-N-CG) neuroblastoma cells	Incubator	1% O_2_	24 h	NA	NA	NA	NA	NA	↓ *Mme* ↓ Neprilysin level & activity	NA	NA	NA
Liu et al. 2016	APP_swe_/PS1_dE9_ mice (3 mo)	Hypobaric chamber	11.1% O_2_	6 h/day for 30 d followed by up to 5 mo normoxia prior to sacrifice	NA	↑ AβPP = C99/C83 ratio	↑ Bace1 (in wt)	↑ Aph1a ↑ Nicastrin ↑ Pen2 = Presenilin-1	↑ soluble & FA Aβ_42_/Aβ_40_ ratio ↑ plaque area & number	↓ Neprilysin	= NFT number ↑ p-tau/tau ratio (at 4 mo)	↓ syn ↓ EM	↓ MWM
